# Scottish Nurses in London

**Published:** 1918-12-21

**Authors:** Ada A. Douglas

**Affiliations:** Commandant. 82 Cadogan Square, Belgravia, S.W. 1.


*To the Editor of* The Hospital.

### Scottish Nurses in London.

*To the Editor of* The Hospital.

Sir,—A Hostel has been opened for Scottish nurses and V.A.D.s on active service at the above residence, kindly lent by the Dowager Lady Renshaw. Active workers on behalf of the Scottish community are aware that many girls passing through London have difficulty in securing accommodation for a short stay in town. It is to remove this obstacle and to provide a comfortable home from home for Scottish girl workers that the Hostel has been started. The work is voluntary, the charges extremely moderate, and the need, owing to the crowded condition of London, is urgent. I shall be grateful if friends will do what they can to advertise the fact that Scottish girls on war work will be made welcome at any time.—Yours sincerely,

Ada A. Douglas, Commandant.

82 Cadogan Square, Belgravia, S.W. 1.

December 17, 1918.

